# Scan, extract, wrap, compute—a 3D method to analyse morphological shape differences

**DOI:** 10.7717/peerj.4861

**Published:** 2018-06-08

**Authors:** Martin Horstmann, Alexander T. Topham, Petra Stamm, Sebastian Kruppert, John K. Colbourne, Ralph Tollrian, Linda C. Weiss

**Affiliations:** 1 Department of Animal Ecology, Evolution and Biodiversity, Ruhr-Universität Bochum, Bochum, Germany; 2 School of Biosciences, University of Birmingham, Birmingham, United Kingdom

**Keywords:** 3D morphological comparison, 3D morphology, Confocal microscopy, Daphnia, Landmark-rare shapes, Confidence ellipsoids, Shape and form analysis, Visualisation of shape alteration

## Abstract

Quantitative analysis of shape and form is critical in many biological disciplines, as context-dependent morphotypes reflect changes in gene expression and physiology, e.g., in comparisons of environment-dependent phenotypes, forward/reverse genetic assays or shape development during ontogenesis. 3D-shape rendering methods produce models with arbitrarily numbered, and therefore non-comparable, mesh points. However, this prevents direct comparisons. We introduce a workflow that allows the generation of comparable 3D models based on several specimens. Translocations between points of modelled morphotypes are plotted as heat maps and statistically tested. With this workflow, we are able to detect, model and investigate the significance of shape and form alterations in all spatial dimensions, demonstrated with different morphotypes of the pond-dwelling microcrustacean *Daphnia*. Furthermore, it allows the detection even of inconspicuous morphological features that can be exported to programs for subsequent analysis, e.g., streamline- or finite-element analysis.

## Introduction

Quantitative analysis of shape and form is critical in many biological disciplines (Adams, Rohlf & Slice, 2004; Slice, 2007; Adams, Rohlf & Slice, 2013; Mitteroecker et al., 2013). Morphological alterations within species often reflect changes in gene expression and physiology and may occur in specific regions as well as in overall morphology, e.g., as context-dependent phenotypic plasticity (e.g., Agrawal, 2001) or as a result of knock-out-mutants during forward or reverse genetic assays (e.g., Gaiano et al., 1996; Golling et al., 2002). Furthermore, cryptic species identified with genetic approaches can be analysed with respect to subtle, but quantifiable morphological traits (Tan et al., 2010), helping to reveal inconspicuous morphological differences. Another application for detecting shape alterations is the analysis of developmental stages, where sites of shape changes or growth can be found by comparing samples at different time points, e.g., in the leaf margins of developing *Arabidopsis* (Bilsborough et al., 2011).

Historically, in morphology research lengths of body parts, i.e., the distance between two points, have been used to measure and compare the evolution and development of different morphologies (e.g., Lagler, 1962). Unfortunately, distances can only capture dimensions and ratios, not underlying shape (Klingenberg, 2016). Furthermore, these measurements, for example when being measured on two-dimensional images, often miss the three-dimensional aspects of the structure, especially when, for example, they are taken on photo images (e.g., Polly, 2003). Furthermore, not every morphological form is characterized by sufficient features to create a dense net of distance measurements that allows to deduce the shape from these measurements in any other than just the simplest way (Zelditch, 2004). In fact, with distances alone, in most cases only lengths, widths or heights of the sample, or parts of it, can be measured.

To describe morphotypes, morphometrics commonly uses explicit morphological landmarks, such as anatomical features, captured within a coordinate system to record not only a set of distances, but the relative arrangement of all landmarks and their interrelations (Mitteroecker & Gunz, 2009; Drake, Coquerelle & Colombeau, 2015; Klingenberg, 2016). For morphological comparisons with geometric morphometric tools, landmarks are fitted, e.g., with a Procrustes analysis (Bookstein, 1993; Dryden & Mardia, 1998). This fit minimizes the squared sum of the landmarks’ distances between the compared morphotypes. This removes differences in position, rotation and size, and allows the comparison of shape (Kendall, 1977; Dryden & Mardia, 1998). When morphotypes are freed from position and rotation by a partial Procrustes analysis the landmark-set still holds differences in size and shape, which we here define as form alterations.

The use of landmarks allows the capture of shape and form in two as well as three dimensions. Especially three-dimensional information is important in many cases, as otherwise alterations not quantifiable in two dimensions are missed. Indeed, the third dimension might be an important explanatory variable, for example in ecological contexts (Cardini, 2014; Openshaw et al., 2016; Buser, Sidlauskas & Summers, 2017). The third dimension can be captured with the help of 3D imaging strategies, during which—if not instantly reduced to certain landmarks—often massive amounts of image data are produced. Use of these data calls for dedicated data handling and modelling strategies (as in, e.g., Martone et al., 2008; Saalfeld et al., 2009).

Such techniques for capturing 3D features include confocal laser scanning microscopy (cLSM), micro-computed tomography (µCT), focused ion beam (FIB) and 3D scanning electron microscopy (SEM), all of which have undergone continuous development in the last decades (Carlsson et al., 1985; Morton et al., 1990; Tafti et al., 2015), leaving the analysis techniques somewhat behind (e.g., Martone et al., 2008). For example, cLSM captures information on a sample’s depth in the inter-slice distance of the generated image stack. In principle, these reconstructions allow detailed computer-based morphological comparisons, provided that suitable tools are available.

Always problematic, even with the application of geometric morphometrics, have been samples that exhibit just few morphologically distinct features (Klingenberg, 2008). Such samples barely offer explicit biologically homologous points, rendering difficult even the use of so-called semi-landmarks, which are defined as landmarks on curves or surfaces connecting morphological features (Bookstein, 1997; Zelditch, 2004). The first solutions to the comparability problem of almost ‘homology-free’ shapes that facilitate the analysis of such datasets have been published in recent years (Boyer et al., 2011; Durrleman et al., 2014; Boyer et al., 2015; Pampush et al., 2016; Pomidor, Makedonska & Slice, 2016). Boyer et al. (2011) and Boyer et al. (2015) solely describe the mathematics behind the alignment and comparison. The other publications offer implementations with a limited graphical user interface, in which the surfaces are loaded and subsequently aligned and compared. Methodologically, Pomidor, Makedonska & Slice (2016) use Symmetric Iterative Closest Point Superimposition for alignment and the Procrustes surface metric to quantify shape differences, while Durrleman et al. (2014) use an atlas and control points for these tasks.

To provide an alternative, semi-automated and Matlab-based approach to these challenges, we introduce a workflow that allows the generation of comparable 3D models for comparison of almost landmark-free morphotypes even with just inconspicuous (i.e., hidden or otherwise disguised) morphological differences. This workflow uses simple steps: (i) *Scan* the sample; (ii) *extract* the 3D surface area, (iii) *wrap* and adjust a grid of explicit points around the outer surface area after arranging the samples in homologous orientation; and (iv) *compute* a superimposition of comparative morphotypes.

In the following, we will introduce these steps in more detail: (i) in our use-case *Daphnia*, confocal image stacks were *scanned* in liquid so that our sample’s physical state is maintained, precluding, for example, drying artefacts accompanying SEM preparations. Depending on the sample and its preservation, any other scanning technique, for example µCT, FIBSEM etc., could work as well. (ii) From the resulting image stacks, we *extract* surfaces using the ‘Marching Cubes’ algorithm (Lorensen & Cline, 1987), producing a triangular mesh comprised of vertices and faces. Such three-dimensional surface- or even volume-renderings have become widespread (Liu, Weaver & Taatjes, 1997; Wei et al., 2012; Barbier de Reuille et al., 2015), and can also be extracted using other algorithms, such as manifold dual contouring (Schaefer, Ju & Warren, 2007). The mentioned vertices are often arbitrarily numbered, preventing direct comparisons of individual vertex positions, i.e., point positions. To enable point-wise comparability, explicit landmarks or alternatively other corresponding points (which are difficult to identify on some shapes) are necessary. Therefore, this is not applicable in the case of landmark-free shapes. Likewise, landmarks dictate the resolution with which shapes can be analysed. (iii) To establish the point-specific comparability required for direct comparisons through homologously numbered and positioned points, we *wrap* the extracted surfaces by projecting two-dimensional silhouettes, inspired by ‘shrink-wrapping’ or the automated wrapping described by Sigal, Hardisty & Whyne (2008). (iv) For the *computational comparison* between individuals and between treatment groups, resulting casts can then be aligned with a Procrustes-fit to find a superimposition that reduces the (partial) Procrustes distance between the samples to a minimum (Gower, 1975; Bookstein, 1993; Dryden & Mardia, 1998). Subsequently, form and shape alterations can be statistically investigated in a point-wise manner, by analysing individual casts. This also allows detection of within-group variance, but also average shapes of different treatments can be compared. We then display alteration in form of heat maps, as published earlier by, e.g., Gunz et al. (2009) and Pomidor, Makedonska & Slice (2016).

We decided to develop and illustrate this workflow using our model organism *Daphnia*, as it provides distinctive morphological variabilities (Fig. S1) (Tollrian & Dodson, 1999; Laforsch & Tollrian, 2004; Weiss, Laforsch & Tollrian, 2012a).

## Methods & Results

The following sections describe the workflow and its results in our use-case *Daphnia* and are divided according to the four steps outlined above: (I) Scan, (II) Extract, (III) Wrap and (IV) Compute.

### Scan—3D capture with confocal imaging on Congo Red stained animals

In our use-case, we performed confocal laser scanning microscopy, and therefore applied fluorescent labelling. Staining of the animals’ integument was carried out with a 0.15% solution of the fluorescence dye Congo Red (Carl Roth GmbH + Co. KG, Karlsruhe, Germany) on a shaker (ELMI Ltd., Riga, Latvia) at 60 rpm for 24 h (Michels & Büntzow, 2010) to ensure even staining. Samples were rinsed three times for at least 10 min with deionised water to remove excess stain. Mounting was performed with ringed sticky tape according to Weiss et al. (2012b).

**Figure 1:**
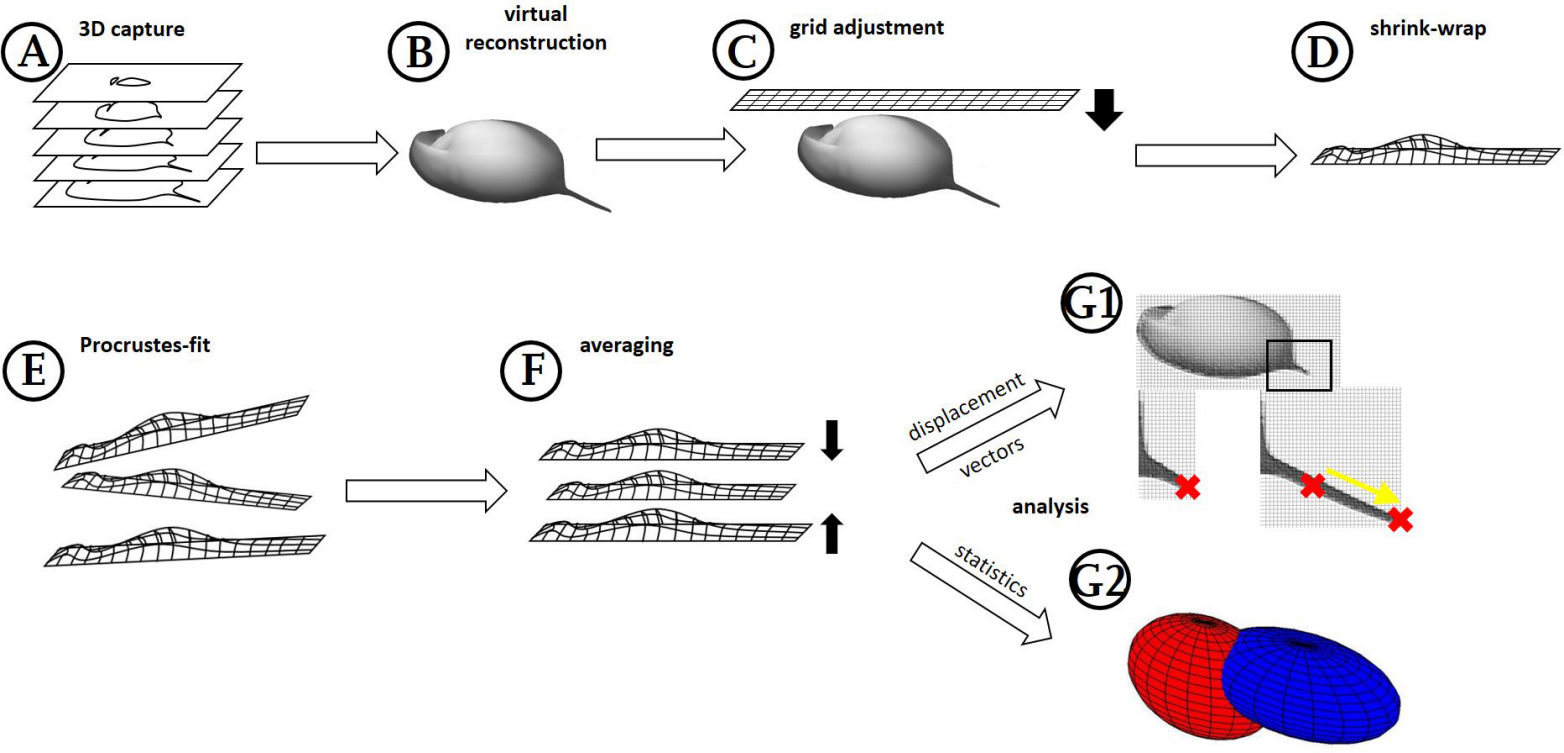
Workflow generation and process. (A) *Z*-stacks/3D scans are acquired from the specimens investigated. (B) Surfaces are extracted (where necessary) in MorphoGraphX. (C) Grids are adjusted to these surface extractions in Blender, based on anatomical fixed points in the outline and a few on the curved surface itself (for details see Fig. 2). (D) By projecting the grid onto the surface extraction, the grid gives a cast of the curvature of the animal. (E) The casts, which are all individually oriented and positioned and therefore a bit different from each other, are (F) aligned with Procrustes-fit using Matlab. The projected grids are then (G1) averaged, and displacement vectors calculated between similar points in both treatments *or* (G2) statistics are performed with Wilcoxon-tests and a subsequent FDR-analysis or ‘confidence ellipsoids’.

Scanning was conducted with a Leica confocal laser scanning microscope (Leica TCS SP5II, Leica Microsystems, Wetzlar, Germany) using 561 nm laser excitation and a detector range of 600–750 nm (Fig. 1A). These settings gave high fluorescence intensities for each acquired stack. Gain was set to 500 V, the pinhole to 70.75 µm and zoom left at 1.0×. Single image stacks were acquired with 400 Hz and saved at 512 × 512 pixels, with a pixel edge length of 3 µm. The resolution in the *z*-direction, which is dependent on the individual slices’ distance, was set to 2.5 µm and produced stacks of approximately 200 slices. If animals were larger than the visual field of a single slice, multiple stacks were acquired with fixed upper and lower limits. Using the stitching function of the microscope’s software (LAS AF 2, Leica Microsystems, Wetzlar, Germany), multiple stacks were composed into one stack. As the confocal lasers were limited with respect to tissue penetration, scanning was conducted for one half of the specimen’s body, as *Daphnia magna* shows a bilateral symmetry. Only intact specimens without any indentations or other shape-influencing alterations were used for the analysis.

### Extract—Virtual reconstruction

The cLSM image stacks were converted to tif-stacks with the Bioformats-Plugin of FIJI (Schindelin et al., 2012), making them compatible with the software MorphoGraphX (Barbier de Reuille et al., 2015). Within MorphoGraphX, ‘Gaussian Blur’, set to a blur rate of 5 µm in each direction, was applied to smooth the intensities. The surfaces of ten undefended and nine defended *D. magna* were extracted along high intensity values using MorphoGraphX’s ‘Marching Cubes Surface’ mesh creation tool, with a cube size of 15 µm, creating surface meshes representing the outermost layer and thus surrounding the volumes of the specimens (Fig. 1B) (Lorensen & Cline, 1987). The algorithm generated triangular meshes consisting of vertices and faces. The threshold for intensity was selected based on each individual stack’s signal intensity, ranging from 500 up to 30,000. The resulting meshes were smoothed with MorphoGraphX’s smoothing tool, not exceeding 1–3 passes to prevent mesh shrinkage. Of course, other algorithms and tools for surface extraction and subsequent smoothing are published and could be used as alternatives, e.g., Schaefer, Ju & Warren (2007). This part of the workflow is only necessary for image capture procedures that have no gridding implemented.

**Figure 2:**
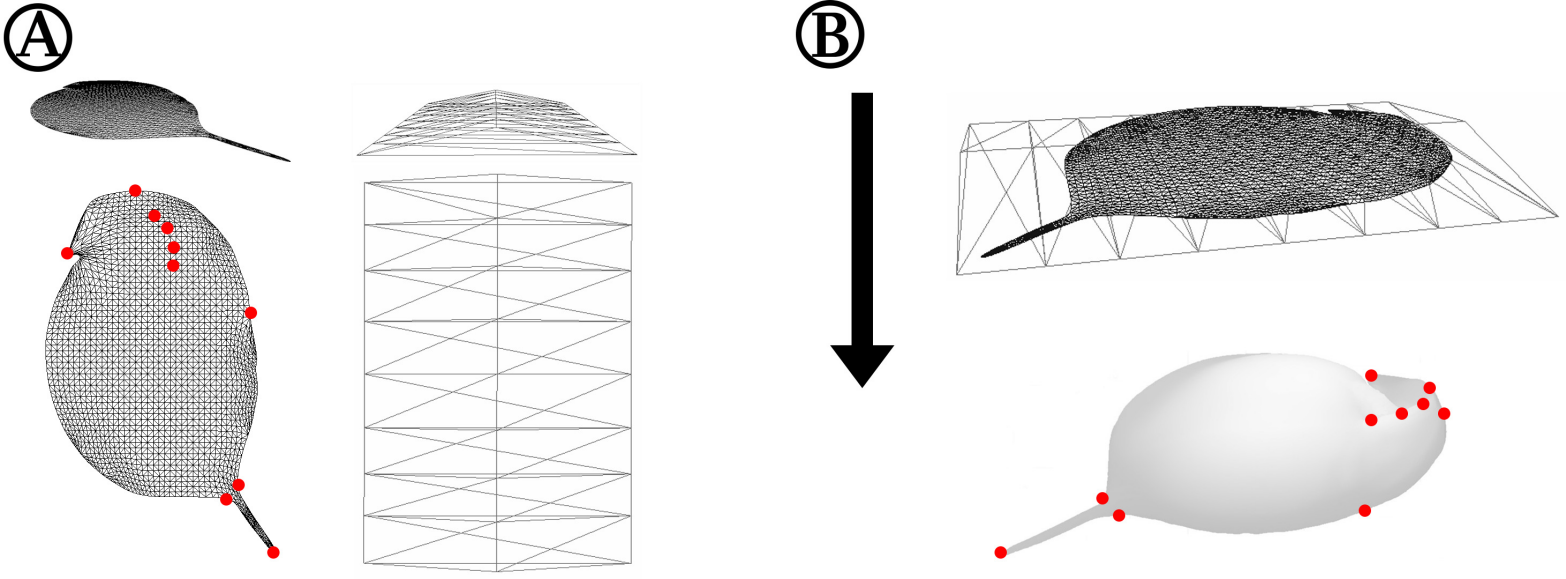
Detailed depiction of the grid adjustment step (Fig. 1C). (A) Detailed representation of the 2D grid that is projected onto our case study organism *Daphnia* in oblique (top left) and top view (bottom left). Some exemplary points of the 2D grid that were used to adjust the grid to landmarks of the 3D mesh are indicated with red dots. The shape of the ‘deform mesh’ (Blender, version 2.73, Blender Foundation, Blender Institute Amsterdam, https://www.blender.org/, 2015) is shown, again in oblique (top right) and top view (bottom right). (B) For adjustment, the deform mesh is arranged around the 2D-grid (top) and elastically bound to it in order to allow the deformation of the fine mesh by moving just a few points of the ‘cage’. Also displayed is the arrangement of the surface mesh exported from MorphoGraphX beneath the deform mesh surrounding the 2D silhouette grid. The arrow indicates the direction of projection of the 2D-grid to produce a cast of the surface mesh. Landmarks of the surface mesh to which the 2D-grid-silhouette is adjusted are highlighted with red dots, matching the corresponding points marked in (A).

### Wrap—Standardisation via grid adjustment, ‘shrink-wrap’ and Procrustes-fit

At this stage, analytical comparison between surface meshes—e.g., shape collations aiming to detect subtle differences between groups of meshes—requires a common coordinate system to be used across all meshes. Coordinate names of the extractions in MorphoGraphX are given randomly in coordinate space, thus preventing a point-by-point analysis when two or more surface meshes are compared. Furthermore, each surface mesh needs to be similarly oriented. We used three landmarks for orientation of the surface meshes and subsequently overlaid them with a 2D grid of about 128,000 points (Figs. 1C and 2A). Grid adjustment was done with respect to recurring features in the animal’s outline and on the 3D surface of our samples (Fig. 2) to fit this highly resolved grid to our samples’ individual forms. In our use-case, such landmarks and semi-landmarks are located at the animals’ tail spine, the vertex of the head, the tip of the rostrum, the carapace indentation in the heart region and points located on the heads’ and carapaces’ folds (Fig. 2). For this adjustment in a standardised position and orientation as well as the grid projection in *z*-direction onto MorphoGraphX-surface-meshes, we used the program Blender (version 2.73, Blender Foundation, Blender Institute Amsterdam, https://www.blender.org/, 2015). We applied the ‘elastic’ adjustment with the tool ‘deform meshes’ (Blender, https://docs.blender.org/manual/en/dev/modeling/modifiers/deform/mesh_deform.html; Joshi et al., 2007) to fit the grid before projecting it (Figs. 1C and 2B). Points of the deform mesh are elastically bound to the grid, hence facilitating and standardising grid adjustment. Thus, adjustments are smoothed in contrast to adjustments based on only a few recurring features. The latter usually gives an edgy form unsuitable for e.g., biomechanical finite element analysis investigations. Subsequently, we projected the adjusted grids onto the original surface meshes by parallel projection along the *z*-axis, giving ‘shrinkwrapped’ casts (https://docs.blender.org/manual/en/dev/modeling/modifiers/deform/shrinkwrap.html). These consist of explicit points with *x*, *y* and *z* coordinates resulting from the explicitly numbered grid (Fig. 1D). Casts can be exported as .obj files (similar to .csv files with headers), and from there are easily imported into Matlab (Matlab R2014b, The Mathworks Inc., Natick, MA, 2015) as one table with all explicit points and their coordinates (Fig. 1E). Points of the grid that did not overlap the surface mesh after ‘shrink-wrapping’ projection, e.g., at parts of the dissected antennae, were eliminated to avoid bias from wrong or lacking projections. All further analyses were carried out within the Matlab environment (see Files S1 and S2).

### Compute—averaging and analysis of results

From casts describing individual morphotypes (reared with or without *Triops*) we created 3D models by averaging the *x*, *y* and *z* coordinates of all explicit points of all casts belonging to the predator- or control-treatment using the Matlab script (see File S1). Subsequently, we compared these models by fitting superimpositions of points of the defended model onto the undefended control model in smallest partial Procrustes distance, using the procrustes()-function (https://de.mathworks.com/help/stats/procrustes.html) (Fig. 1F) (Gower, 1975; Bookstein, 1993; Dryden & Mardia, 1998). The Procrustes-fit prevents the false detection of shape modulation due to dissimilar positioning in 3D-space (Fig. 1F). Generally, this procedure can also make size modifications that shrink and stretch the casts to fit each other. Here, however, size may be an important factor for ecological morphotype comparisons, and so the size adjustment was not conducted. As the obtained result is not shape by definition (Kendall, 1977; Dryden & Mardia, 1998), we refer to these results as alterations of form. Nevertheless, we also tested the comparison of morphotypes with enabled size-adjustment (File S1, Fig. S2), giving shape alterations (reviewed in Klingenberg, 2016).

**Figure 3:**
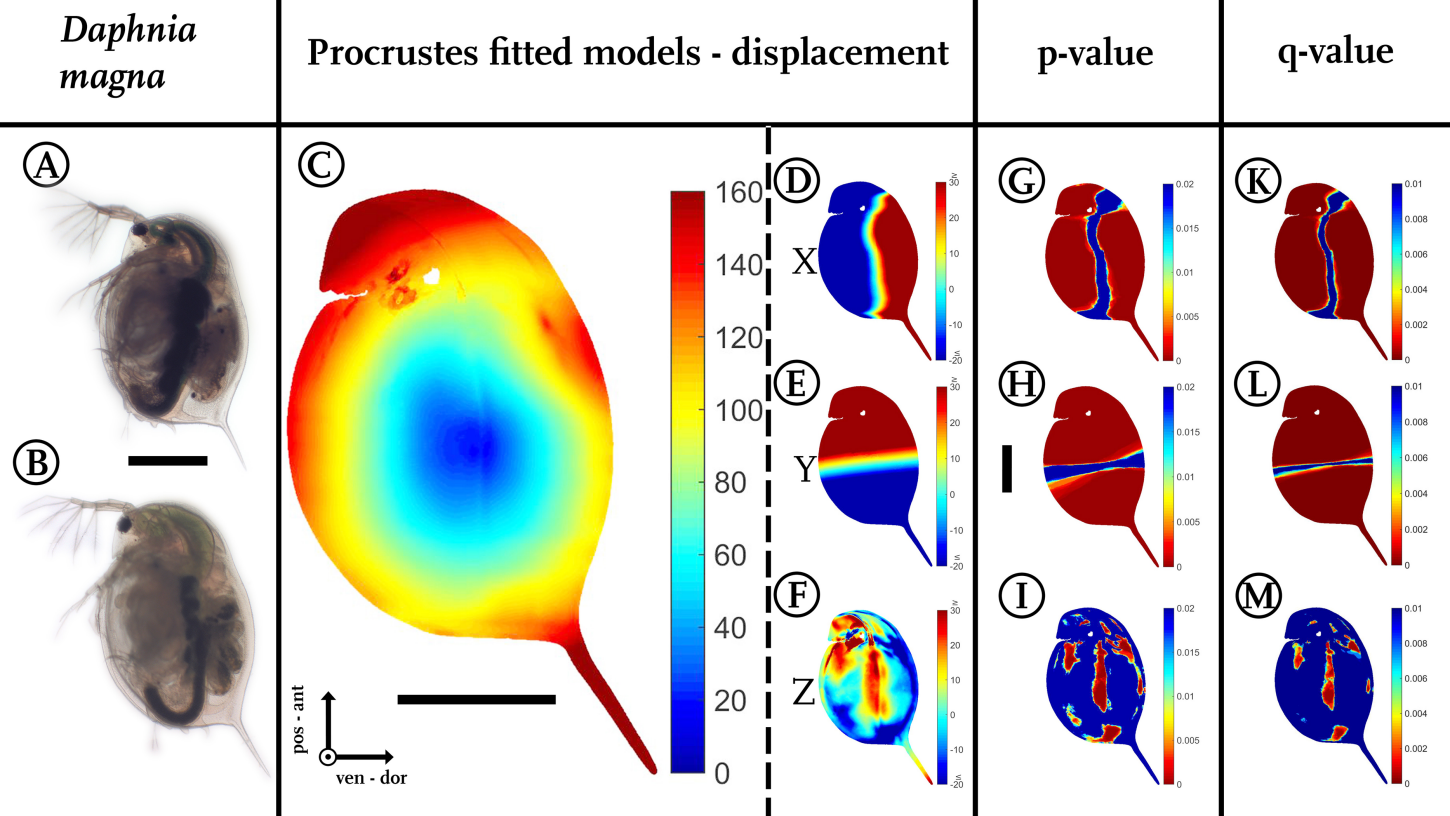
Analysis of model comparison. (A) Photograph of an undefended *D. magna*. (B) Photograph of the defended morphotype of the same species. (C) Overall displacement vectors, displayed as a heat map. Shades of red indicate long displacement vectors, shades of blue small displacement vectors. (D) Displacement in *x* direction, (E) displacement in *y* direction. (F) Displacements in *z*-direction. Blue colours in (D–F) represent alterations in the negative direction of the respective axis, while red shows positive alterations along the respective axes. Shades of yellow to white indicate little or no shifts along the respective axis. (G) Wilcoxon-test performed for the *x* coordinates, (H) Wilcoxon-test performed for the *y* coordinates (I) Wilcoxon-test performed on the *z* coordinates. Wilcoxon-tests in (G–I) were conducted taking into account all samples of the two treatments (*n*_undefended_ = 10, *n*_defended_ = 9). Colours ranging from red to green mark *P*-values smaller than 0.01, with red giving highly significant results. (K) *Q*-value plot for the *x*-coordinates (L) *Q*-value plot for the *y*-coordinates (M) *Q*-value plot for the *z*-coordinates. Note the colour scaling difference in (K–M). Colours ranging from red to green mark *q*-values smaller than 0.005. Scale bar for (A) and (B) is given between them, scale bar for (D–M) is given next to (H), all scale bars 1 mm. ant, anterior; pos, posterior; ven, ventral; dor, dorsal.

Subtraction of coordinates (in *xyz*-direction) of the undefended mean coordinate points from the adjusted coordinates of the defended mean provides a set of absolute displacement vectors for each semi-landmark point used to construct the grid-mesh models (Fig. 1G1). With the application of the Pythagorean theorem we determined the magnitude of the straight vector between the respective points on the two models by the hypotenuse’s length, with translocations along the coordinate axes being the required catheti (Fig. 3). Therefore, for each coordinate axis these point translocations can be calculated separately and displayed in heat maps. Heat maps of the acquired overall point translocations (Fig. 3C) indicate strongest deformations in shades of red and weakest deformations in shades of blue. Displaying the changes along the various coordinate axes, red indicates changes in a positive direction along the respective coordinate axis, blue indicates displacement in a negative direction on the respective coordinate axis (Figs. 3D–3F). The method maintains actual specimen size so that the length of the displacement vectors, either in overall point translocation plots (Fig. 3C), or along the coordinate axes (Figs. 3D–3F) is given in micrometres.

In our use-case *Daphnia*, we found shortest displacement vectors in the centre of the specimen. Point translocations gradually increase towards the outer limits, indicated by shades shifting from yellow to red (Fig. 3C). We found displacement vector lengths in the range of 20–50 µm in the centre, while the body periphery is translocated by 100–140 µm. In particular, the tail spine, the head capsule and the dorsal region around the heart are coloured dark red, indicating displacement vectors of over 160 µm in length.

Dorsal parts are shifted dorsally (shades of red/positive), ventral parts are shifted ventrally along the *x*-axis (shades of blue/negative, Fig. 3D). In between, an intermediate zone (shaded yellow to white) with minor or no shifts is visible. Likewise, anterior regions are shifted anteriorly (red/positive), while posterior regions are shifted posteriorly on the *y*-axis (blue/negative, Fig. 3E). Similarly, the increase in lateral width is marked in dark red (Fig. 3F).

For statistical analysis, two-sided Wilcoxon signed rank sum (U-)tests (ranksum()-function; https://de.mathworks.com/help/stats/ranksum.html; Fig. 1G2) were calculated using the coordinates of the individual casts from both treatments to determine differences between all morphotypes from two independent groups (Figs. 3G–3I). For model normalization within all three dimensions, individual casts were superimposed again using the Procrustes fit without size adjustment (Fig. 1F). This ensured optimal orientation for statistical analysis. Point-to-point and direction-wise analysis of defended and undefended *xyz*-data was conducted using the Wilcoxon-test in Matlab (Matlab R2014b, The Mathworks Inc., Natick, MA, 2015). Therefore, a test was calculated for every point’s *x*, *y* and *z* coordinates. The compared casts consist of over 120,000 points describing the form. Accordingly, more than 360,000 Wilcoxon-tests were calculated automatically, using the Matlab-script (File S1).

Our script colours body regions in shades from red to green to delimit significant differences with *P*-values smaller than 0.01 (two-sided Wilcoxon-test, *n*_undefended_ = 10, *n*_defended_ = 9; Figs. 3G–3I). Shades from yellow to blue delimit *P*-values above 0.01, indicating no significant changes in form. We found 105,191 points on the surface to differ significantly for the *x*-dimension, 114,596 for the *y*-dimension, and in *z*-dimension 22,365 points showed a *p*-value lower than 0.01.

Corrections for multiple testing for all three dimensions are provided by the false discovery rate (FDR) approach described by Storey & Tibshirani (2003), implemented in Matlab as mafdr()-function (https://www.mathworks.com/help/bioinfo/ref/mafdr.html). Applying this function with default parameters gave *q*-values for every tested point (Figs. 3K–3M) and values for *π*_0_ and *π*_1_. The *π*_1_, calculated for each dimension in our use-case, gives a lower bound on the proportion of features that truly follow the alternative hypothesis (Storey & Tibshirani, 2003). In our use-case the majority of the calculated tests are indeed significantly different. For the *x*-dimension we estimate 93% of the tests correctly reject the null hypotheses, in the case of the y dimension this number is even 97% and for the *z*-dimension we can estimate at least 67% of the tests to reject the null hypothesis correctly.

A colour-coded plot of the *q*-values onto the 3D-model reveals most regions as statistically significant with *q*-values below 0.005.

The direct *p*-value- *q*-value-plots can be found in the supplementary material (Fig. S3).

**Figure 4:**
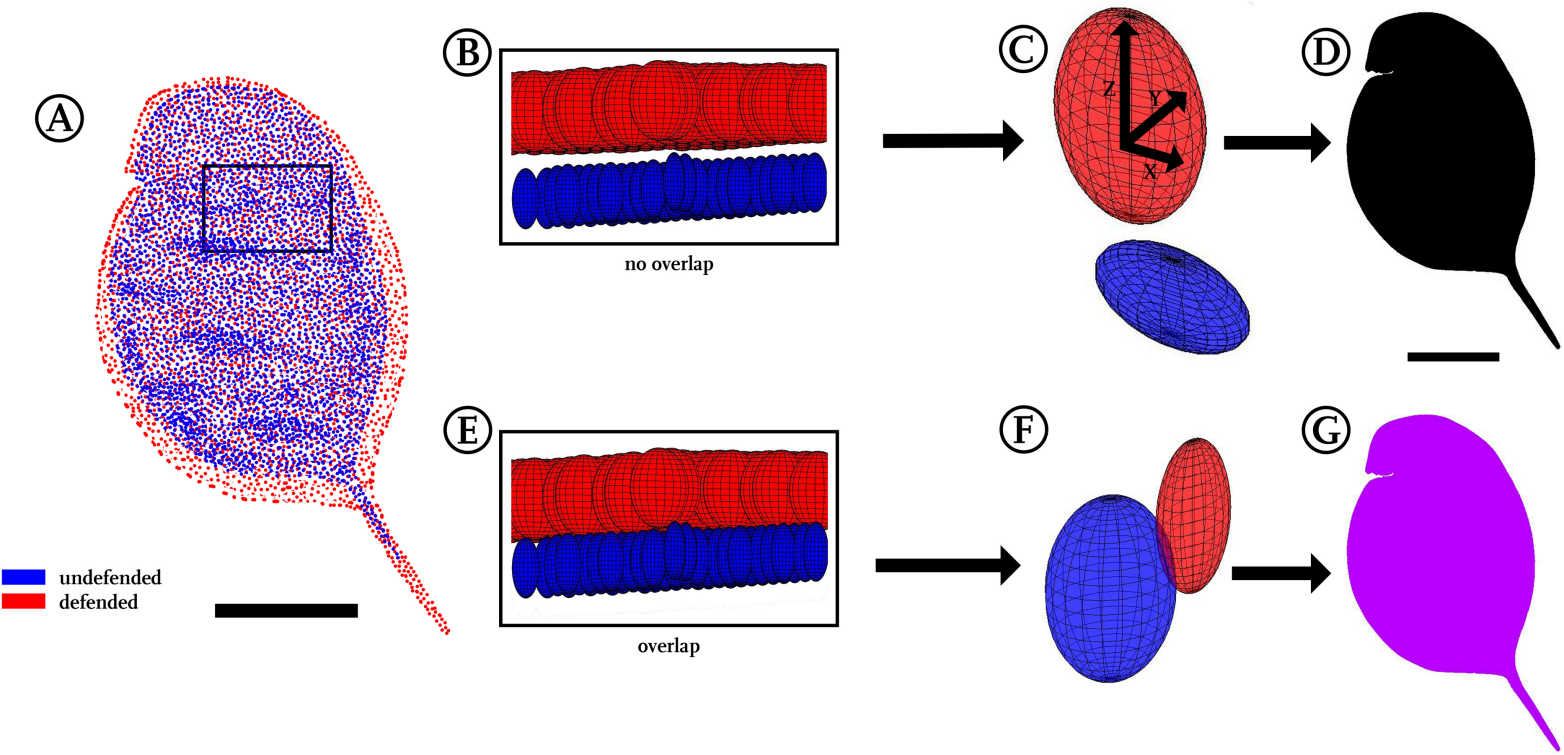
Confidence ellipsoid analysis. (A) Illustration of the average point positions in the undefended (blue) and defended (red) treatment. (B) Detail of a region next to the head, illustrating confidence ellipsoid positions of both treatments. The overlap or non-overlap of ‘confidence ellipsoids’ can be distinguished. Note the much more voluminous ellipsoids of the defended treatment (red), hinting at the increased variance in this group. (C) Detail of a single confidence ellipsoid pair with no overlap which is the only case occurring in *D. magna*. The non-overlap indicates that major differences can be found between the average positions of all points with relatively small variance. Confidence ellipsoids were determined centred on the average point position and surrounded by a volume spanned three-dimensionally by the 95%-X-Y-Z confidence intervals of each point pair as radii around them. (D) The non-overlap situation present in this study is indicated with black colouring in regions of non-overlap. (E) As a test of this way to visualise statistics, we permutated the treatments, resulting in 50% of the original shapes interchanged between the treatments. (F) This led to overlap of all respective ellipsoids, shown here exemplary on a single confidence ellipsoid pair. (G) Regions of the body found to show overlap were therefore coloured purple, inspired by the result of mixing red and blue representing the original treatments. All scale bars 1 mm.

For a three-dimensional statistical analysis, we calculated the 95%-confidence intervals for each point’s *x*, *y* and *z* coordinates, which, applied as radii, span a confidence ellipsoid for all points of the two groups (Figs. 1G and 4C). We therefore used the Ellipsoidal Toolbox (Kurzhanskiy & Varaiya, 2006), especially the ellipsoid()- and ellipsoid.distance()-function in Matlab. The centre of each ellipsoid is defined by the average coordinates of a point across all individuals, as described above in the preparation of displacement vectors. Subsequently, the confidence ellipsoids of all points from each model are analysed for their overlap. Overlapping ellipsoids and their radii provide a better idea of what kind of alteration is occurring rather than a simple direction-specific *p*-value analysis (Nakagawa & Cuthill, 2007). No confidence ellipsoid overlap suggests statistical differences between the groups and is displayed in black (Fig. 4D), while regions of overlap are displayed in purple (Fig. 4G). We found a fully black model in the analysis of our *Daphnia* use-case. Testing animals of the same group against each other as well as randomly permutating both groups led to overlap in almost all regions (Fig. S4).

## Discussion

We have proposed a workflow that can detect subtle differences in morphology between morphotype groups. As an illustration we used two context-dependent morphologies of *D. magna*, which is known to exhibit predator-induced differences in morphology (Rabus & Laforsch, 2011; Rabus et al., 2012). Superficially, animals of both treatments are similarly shaped, rendering the detection of differences through visual inspection difficult. Earlier approaches found measurable differences between pre-defined inter-landmark distances and describe morphological defenses in *D. magna* (Rabus & Laforsch, 2011; Rabus et al., 2012). With spatially high-resolution models of the animals’ forms, we were able to reproduce their results. Moreover, we found an overall change in morphology in the *z*-direction. We were able to validate that the observed changes are not only due to uniform body growth in all dimensions.

### *Daphnia* as use-case

By comparing the translocations between the dense nets of respective points of both treatments, our approach allows visualisation of alterations by colour-coding of respective regions (Figs. 3, 4D and 4G). Such heat maps were used for example by Pomidor, Makedonska & Slice (2016) to visualize the between-sample variation. With these heat maps, we found that *D. magna* shows a different morphotype when exposed to a predator like *Triops*. We observed an increase in overall body size across all axes (Fig. 3C). In contrast to measuring just distances without any information on the *z*-direction on pictures of the samples, our application offers the ability to determine micrometre-scale shape changes in high resolution as we can compare thousands of surface points between morphotypes.

In contrast to alterations of the outline, alterations in the *z*-dimension are more difficult to detect in bilaterally flattened organisms. In the case of our model system *Daphnia*, only differences in the distance between the fornices have been detected in the *z*-dimension so far (Rabus et al., 2012). However, measuring just one distance in lateral width does not reflect the overall *z*-direction morphology and certainly does not represent a complete description of a morphotype. We were able to determine additional changes of form in the *z*-direction (Figs. 3F and 3I), i.e., a locally enhanced or decreased lateral width. We here find that the morphotypes vary 20–30 µm in lateral width in the centre. By applying the size-adjusting Procrustes fit, almost all alterations disappear. Therefore, here the main reaction to the presence of the predator is an increased body size.

We used the FDR-approach as a correction for multiple testing (Storey & Tibshirani, 2003). This correction revealed a very high proportion of the conducted tests to correctly reject the null hypotheses. The *π*_0_ values, which give a conservative proportion of the null *p*-values, are very small (*x*-dimension: 0.0671, *y*-dimension: 0.0278, *z*-dimension: 0.3273), indicating that 93%, 97% and 67% (*x*-/*y*-/*z*-dimension) of the features found are different between both morphotypes. Also, the *q*-value evaluation of the *p*-values reveals that at a *p*-value threshold of 0.05, the *q*-values range from about 0.01 to 0.03, depending on the respective dimension. This means that in our use-case comparison only 1–3% of the tests with *p* ≤ 0.05 are false positives.

To compare point positioning in a 3D manner, we plotted 95%-confidence ellipsoids that show the overall position changes between both morphotype models. No overlap between the confidence ellipsoids of the two treatments uncorrected for size during the Procrustes-fit could be found among all respective pairwise comparisons, demonstrating a subtle yet remarkable difference between the investigated treatments. Therefore, the approach presented herein not only allowed the conversion of whole 3D surfaces into models, but also facilitated the detection of small differences between morphotypes that are otherwise hard to detect. At the same time this confidence ellipsoid plot (Fig. 4D) is a compression of the data derived in terms of average point position and displacement vectors, as it yielded good impression of the position of shape/form and/or size alterations and their statistical significance. Additionally, plotting confidence ellipsoids region-wise allows comparisons of the volumes and dilatations of these ellipsoids, which helps to get impressions of the difference between morphotypes and variances within each group with respect to the axes (Figs. 4B and 4E).

We here applied the partial Procrustes fit without size-adjustment for superimposition in smallest partial Procrustes distance, since size seemed to be the major alteration between morphotypes, especially due to the ecological background. As described, our workflow also allows to perform a full Procrustes analysis. We used this ‘classical’ Procrustes analysis with size-adjustment to exclude size effects, extracting just shape. Nonetheless, these data can contain a component of size-related shape variation, for example differential growth rates of body structures, i.e., allometry. To identify such, multivariate regressions and principal component analyses need to be conducted (for a review on allometry see Klingenberg, 2016). The full Procrustes analysis revealed only minor alterations of shape, mainly in the region of the head capsule’s dorsal area and the tail spine (Fig. S2). This verifies most alterations found centrally are due to an increase in size. In other circumstances than our use-case, size-adjustments may be useful, for example in developmental studies where the overall growth hides smaller alterations in shape. Furthermore, one can imagine studies of, for example, the development of leaves of *Arabidopsis* that can be investigated for regions of disproportional growth.

### Advantages and applications

Here, we provide an approach designed to be generally applicable. We furthermore expect this approach to be transferable to other species as well as other measurement scales. These features also apply to other already published approaches to “homology-free” morphometrics (Polly, 2008b; Pomidor, Makedonska & Slice, 2016). Previous approaches used algorithms to superimpose surface extractions; e.g., via the first two principal component axes (Pomidor, Makedonska & Slice, 2016) or via conformal geometry and optimal mass transportation (Boyer et al., 2011). Our workflow requires manual adjustment, which positions the animals in a comparable orientation. Therefore, this step is analogous to the automated orientation presented in the mentioned publications. We have not automated this step, even though it is comparatively time consuming. We believe that manual editing avoids bias resulting from insufficient superimposition based on the very rare landmarks on some shapes.

Furthermore, an advantage of our workflow compared to e.g., the automated technique of Pomidor, Makedonska & Slice (2016) is the high density and quite even distribution of comparable grid points, which avoids giving inappropriate weight to certain regions during the Procrustes fit, especially since semi-landmarks add less information compared to landmarks (Zelditch, 2004). Similarly, in the case of missing data, our workflow is more resistant to bias based on missing data of the ‘prototype’, the morphotype model that serves as base for all Procrustes fits. Missing data do not affect morphotype alignment, as no nearest neighbour algorithm is applied as in Pomidor, Makedonska & Slice (2016). Regions that are missing in the prototype are omitted from the analysis. Therefore, the prototype should preferably be a representative specimen, as recommended in most analyses, e.g., (Durrleman et al., 2014).

Gunz et al. (2009) and Polly (2008a) also use point grids to create semi-landmarks. While Polly (2008a) fits a grid onto a shape, Gunz et al. use a thin plate spline approach. Yet, both publications lack downstream comparative analysis between morphotype groups. The eigensurface analysis uses semi-landmarks on the morphotype’s outline and on a central axis that spans a feather-like net of equidistant semi-landmarks on the investigated surface (MacLeod, 2008; Sievwright & MacLeod, 2012). This produces a consistent number of intrarib nodes, which can then be compared in positioning. In the case of large morphotypic differences in the eigensurface and in our approach, semi-landmarks may not be projected on biologically homologous positions, but for detection of generalized form/shape change this might not be an important consideration. Nevertheless, tackling this problem using semi-landmarks as in both latter approaches is one unique way to describe the form/shape of almost landmark-free samples (Klingenberg, 2008; Pomidor, Makedonska & Slice, 2016).

In our approach, all steps are isometric, i.e., the originally captured size can be conserved through all steps of analysis and deformations can be plotted in absolute values. This facilitates evaluation of morphological alterations, e.g., in an ecological context as it is appropriate in our case study. Alternatively, studies on knock-outs of, for example, *Drosophila* or zebrafish embryos, may aim to detect just such size differences in detail (Prpic & Posnien, 2016, and references therein). Thus, the size-adjustment during Procrustes analysis needs to be performed depending on the questions asked in the respective study. Here, by use of both size-adjusted and non-size adjusted data, we were able to detect differences both in size (Fig. 3C), and in specific shape (Fig. S2) between two ecological morphotypes, thus morphology can be studied in the absence of prior assumptions about where and how shape changes occur.

In addition to morphotype comparisons, within-sample analyses can also be conducted, revealing differences in variance of body regions within a single treatment group (like Pomidor, Makedonska & Slice, 2016).

We have shown that our workflow can detect size and shape differences in almost landmark-free samples. We anticipate that this workflow will be applicable in a wide range of species across different kingdoms of life, including morphotypes that lack obvious features that can be analysed for specific characteristics. Also, as long as a few explicit morphological points (landmarks) exist and surface points are *‘wrappable’* by parallel projections along an axis, describing shapes with our workflow should be feasible. Given this prerequisite, adjustment to any critter of choice may be conducted with the supplied Matlab script (File S1: https://github.com/HorstmannM/3Dshape/blob/master/completeDataProcessing_magna_supplement.m// File S2: https://doi.org/10.6084/m9.figshare.5144032). This script is designed for a complete analysis including an automated data-download from an online depository (https://doi.org/10.6084/m9.figshare.5331319). The *computations* allow the analysis of morphological alterations with various approaches. That way, our method may help to resolve species complexes delineated only by complicated or obscure morphological features, such as the challenging kinship relations of diatoms (Round, Crawford & Mann, 1990). As data source we used confocal data processed with MorphoGraphX, but based on the data type after processing, which is 3D surface meshes, we expect that µCT datasets or other 3D-reconstructing techniques are also suitable input sources.

The confidence ellipsoid approach we introduced here for comparisons was found suitable as summary of form and shape alterations occurring between our treatments by visualising statistical significance for all body regions in just a single plot. This is especially interesting in a three-dimensional biological context, as typical *p*-value based statistical tests (Student’s *t*-test, Wilcoxon-test) have frequently been criticised especially in the context of multiple testing scenarios as this one, rendering confidence intervals an interesting alternative (Poole, 1987; Nakagawa & Cuthill, 2007). Furthermore, we tested the reliability of this approach by permutation of investigated samples, meaning a multiple exchange of 50% of the casts between both treatments. This led to a complete overlap of all confidence ellipsoids (Figs. 4E and 4G), supporting the validity of the confidence ellipsoid overlap as a statistical test for differences in form and shape.

Regarding the output of our workflow, the averaged point clouds created for investigated treatments are exportable from Matlab to text files allowing CAD-modelling, streamline- or finite element analysis (FEA) calculations. Alternatively, these analyses can be conducted within the Matlab environment (https://de.mathworks.com/discovery/finite-element-analysis.html). The most common outputs are comma separated values, tabulator separated values or Excel files. Necessary remeshing of these point clouds can be performed in Blender (http://www.blender.org, 2015), MeshLab (Cignoni et al., 2008) or with the Shrink-Wrapped Boundary Face algorithm (Koo et al., 2005), covering the point-cloud models with a mesh. This is an elementary step for further processing of the data, since FEA for 3D-models is dependent on volumes or at least surfaces. Exporting surfaces or point clouds into analysis tools like Amira is also possible. Therefore, a vast array of downstream investigations is possible, e.g., biomechanical or streamline analyses of the compared forms.

## Conclusions

The workflow we presented adds to the arsenal of tools to measure and compare 3D morphologies, based on a representative set of samples. We found that it is useful in comparing almost landmark-free shapes like our use-case organism *Daphnia*. The resolution of the resulting models is adjustable to the needs of the user, but limited by the resolution of the initial scans. Furthermore, not only shape, but also size can be maintained, allowing comparison of the position of points between individuals and/or between and within groups. The detected differences can be plotted as heat maps to visualise shape changes. Additionally, collations can be calculated with various analyses and statistical comparisons. Applying the functionality of computing average models for different groups can simplify downstream applications such as biomechanical or streamline analysis, as it reduces distortion of the results by individual variability (seen in clonal and non-clonal organisms). In summary, this workflow uses new combinations of existing methods for shape analysis and approaches for qualitative and quantitative scoring. This extends the existing set of methods with another powerful 3D approach for detection and visualization of alterations of shape and form. From its results hypotheses for further investigations can be generated.

##  Supplemental Information

10.7717/peerj.4861/supp-1 Figure S1 Light microscopic comparison of undefended (A) and defended (B) *D. magna* K34Q (A) The undefended morphological state is mainly characterised by a comparatively short tail spine, while (B) the one of defended animals is elongated and inserts on an almost circular body outline. Apart from these alterations, no other differences between the shapes can be detected with usual imaging techniques. In (B) the directions respective to the animal’s body are indicated. Scale bar = 2 mm.

10.7717/peerj.4861/supp-2 Figure S2 Overall displacement, size adjusted By applying a Procrustes-fit with adjustment of size, most alterations, as the ones visualised in Fig. 3C, disappear. Therefore, the true changes of shape become obvious, which are mainly located at the tail spine, which is elongated non-proportionally to body length in defended specimens. Further shape alterations of minor magnitude occur in the dorsal area of the head capsule, namely the region of the heart as well as dorsal-anterior body margin of the head capsule.

10.7717/peerj.4861/supp-3 Figure S3 *Q*-values against their respective *p*-values Given are *p*- *q*-plots for the *x*- (A), *y*-(B) and *z*-dimension (C). Figure S3 (D) gives an exemplary *λ*–*π* (*λ*)-plot for the *z*-coordinates, calculated with Matlab, to determine *π*_0_ and therefore indirectly *π*_1_, which is the lower bound on the proportion of test decisions correctly following the alternative hypothesis.

10.7717/peerj.4861/supp-4 Figure S4 Confidence ellipsoid analysis of permutated treatments Displayed are the results of the usual confidence ellipsoid analysis, conducted with the natural treatments leading to complete non-overlap of all the confidence ellipsoids (black clouring) (A). Figures (B) to (D) show three random permutations among the samples, each keeping the number of animals in the treatments constant. All permutated tests show no overlap (purple colouring).

## Additional Information and Declarations

### Data Availability

The following information was supplied regarding data availability: GitHub: https://github.com/HorstmannM/3Dshape; Horstmann, Martin; Topham, Alexander T.; Stamm, Petra; Kruppert, Sebastian; Colbourne, John K.; Tollrian, Ralph; Weiss, Linda C. (2018): Workspace-file containing the values calculated for the variables used in MatlabScript.m. figshare. Dataset. DOI: 10.6084/m9.figshare.5144032.v3; Horstmann, Martin; Topham, Alexander T.; Stamm, Petra; Kruppert, Sebastian; Colbourne, John K.; Tollrian, Ralph; Weiss, Linda C. (2018): Original reconstructions from clsm-scans after wrapping the adjusted grid. figshare. Fileset. DOI: 10.6084/m9.figshare.5331319.v1.
