# Supplementary figures and images for: Scan, extract, wrap, compute—a 3D method to analyse morphological shape differences
Source: PeerJ. 2018 Jun 8;6:e4861. doi: 10.7717/peerj.4861 (PMC5995102; doi:10.7717/peerj.4861)

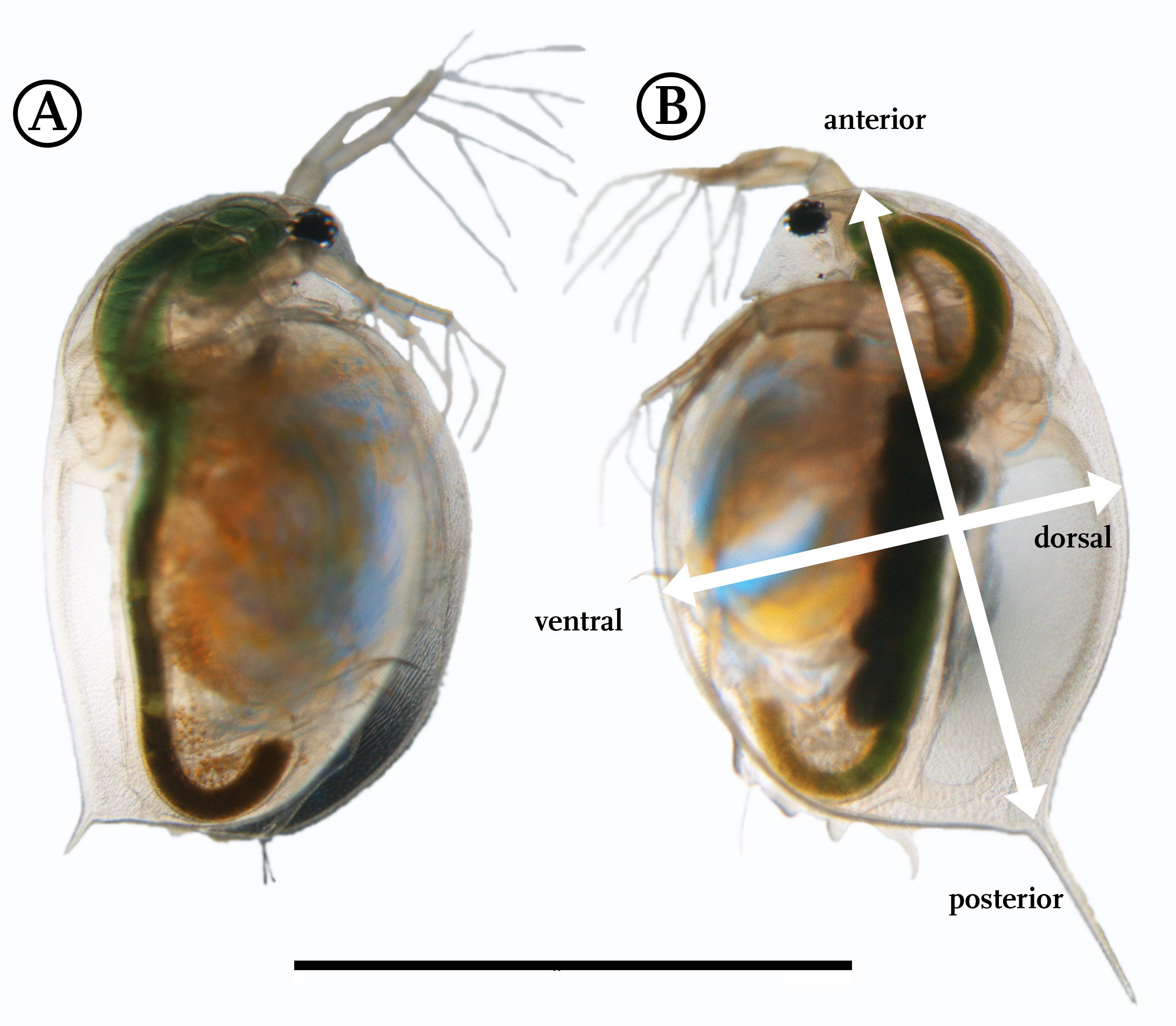

Supplement: Figure S1 — (A) The undefended morphological state is mainly characterised by a comparatively short tail spine, while (B) the one of defended animals is elongated and inserts on an almost circular body outline. Apart from these alterations, no other differences between the shapes can be detected with usual imaging techniques. In (B) the directions respective to the animal’s body are indicated. Scale bar = 2 mm. [file peerj-06-4861-s001.png]

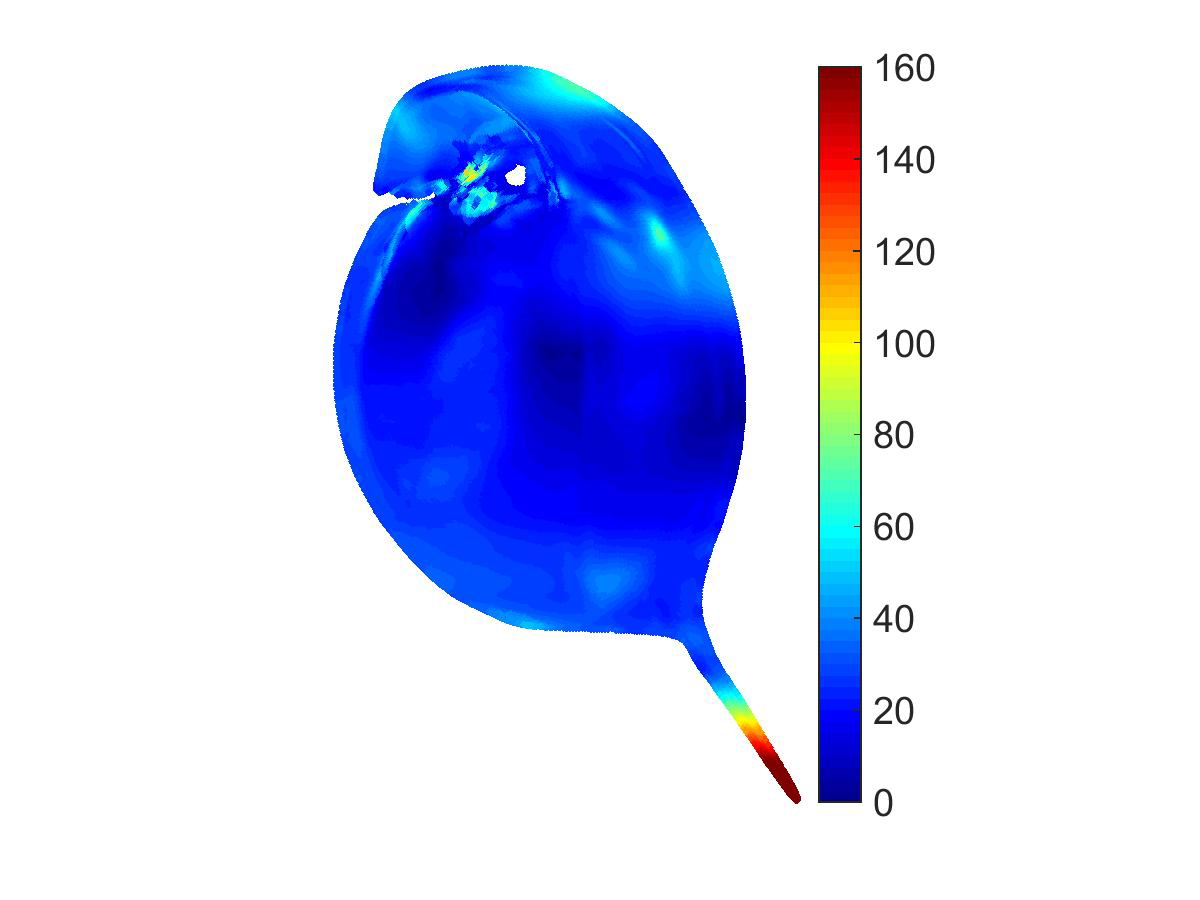

Supplement: Figure S2 — By applying a Procrustes-fit with adjustment of size, most alterations, as the ones visualised in Fig. 3C, disappear. Therefore, the true changes of shape become obvious, which are mainly located at the tail spine, which is elongated non-proportionally to body length in defended specimens. Further shape alterations of minor magnitude occur in the dorsal area of the head capsule, namely the region of the heart as well as dorsal-anterior body margin of the head capsule. [file peerj-06-4861-s002.png]

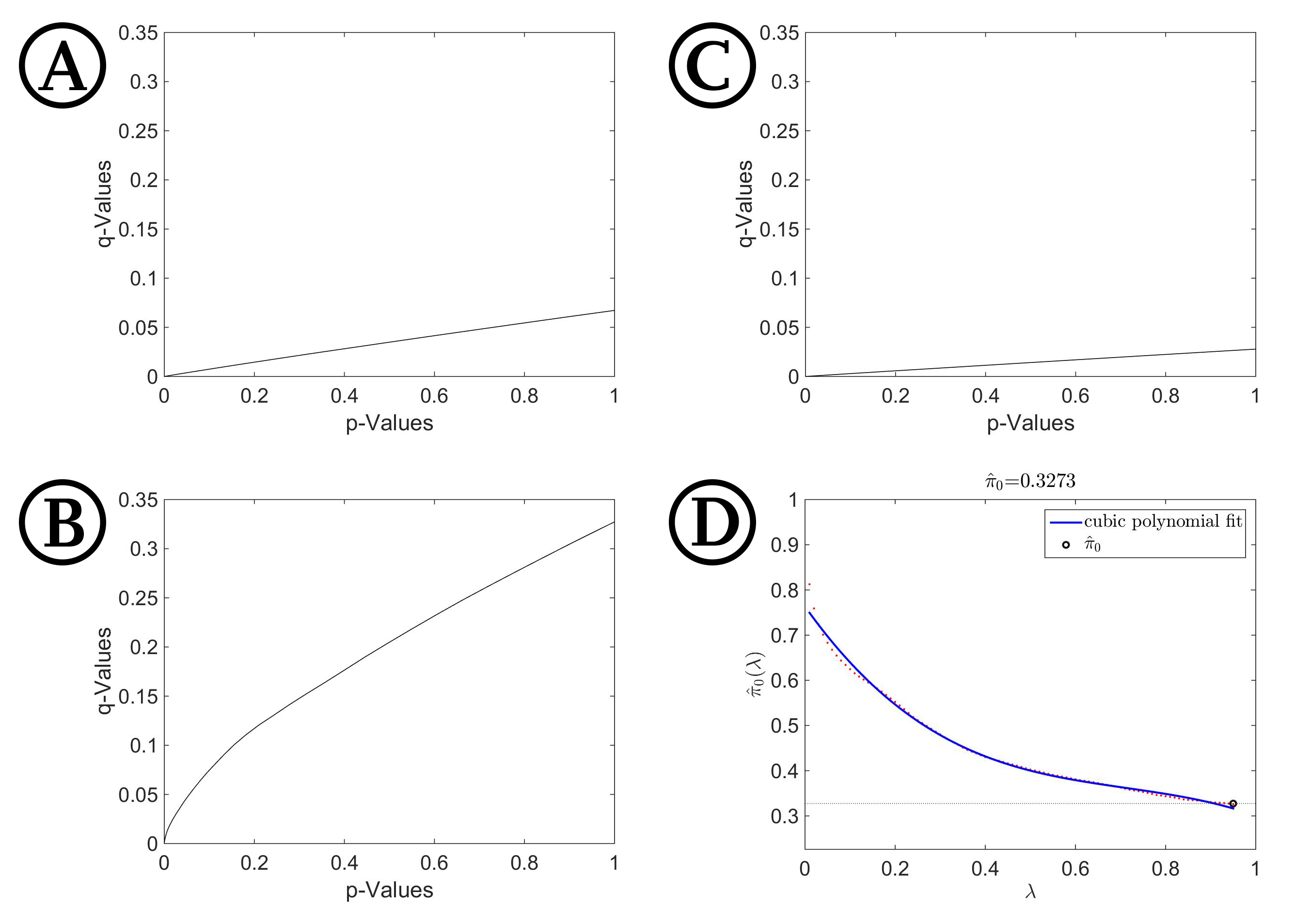

Supplement: Figure S3 — Given are p- q-plots for the x- (A), y-(B) and z-dimension (C). Figure S3 (D) gives an exemplary λ–π (λ)-plot for the z-coordinates, calculated with Matlab, to determine π0 and therefore indirectly π1, which is the lower bound on the proportion of test decisions correctly following the alternative hypothesis. [file peerj-06-4861-s003.png]

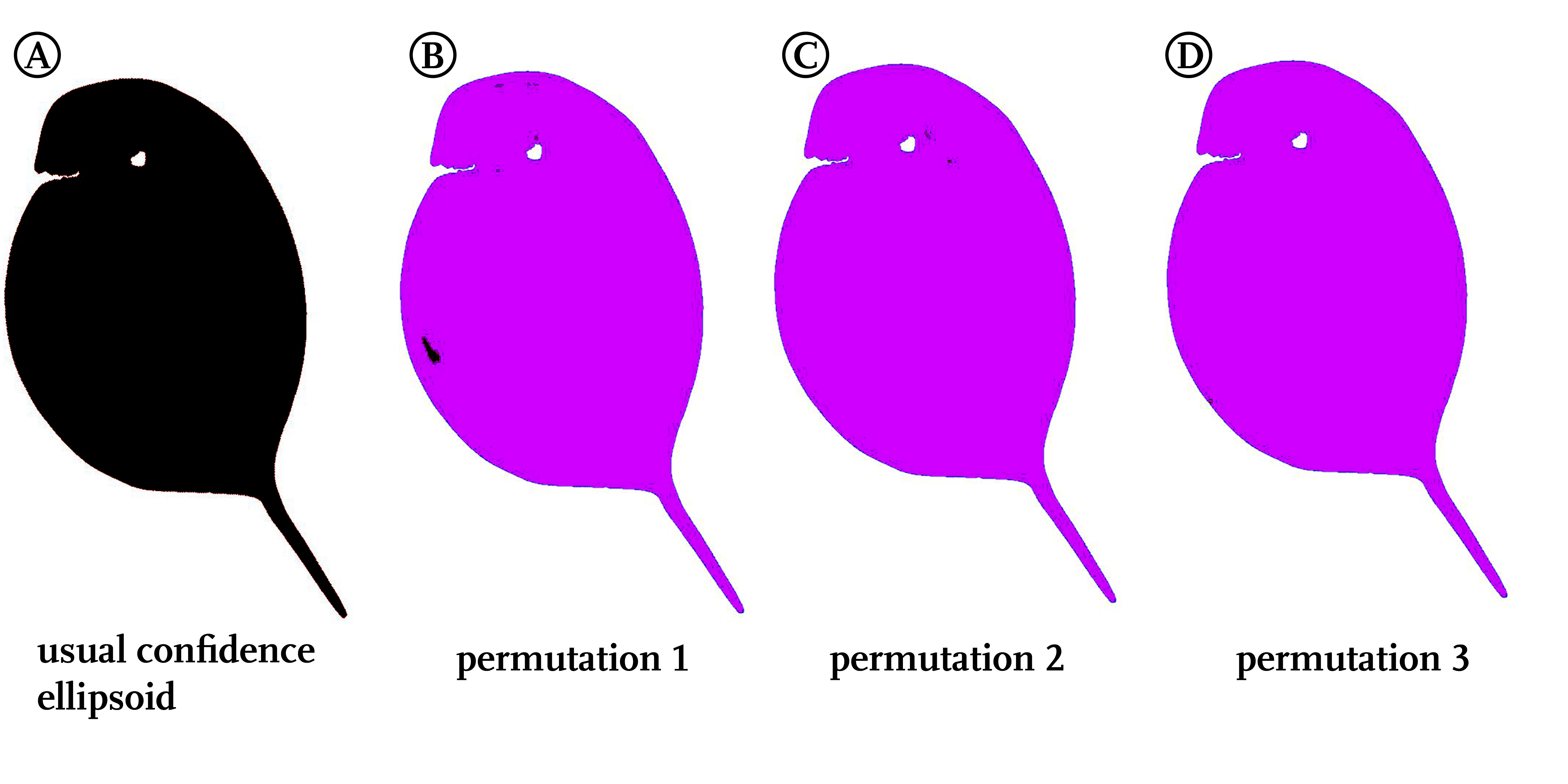

Supplement: Figure S4 — Displayed are the results of the usual confidence ellipsoid analysis, conducted with the natural treatments leading to complete non-overlap of all the confidence ellipsoids (black clouring) (A). Figures (B) to (D) show three random permutations among the samples, each keeping the number of animals in the treatments constant. All permutated tests show no overlap (purple colouring). [file peerj-06-4861-s004.jpg]
